# Supplementary material for: Center backs work hardest when playing in a back three: The influence of tactical formation on physical and technical match performance in professional soccer
Source: PLoS One. 2022 Mar 17;17(3):e0265501. doi: 10.1371/journal.pone.0265501 (PMC8929644; doi:10.1371/journal.pone.0265501)
Supplement: S1 Table — (DOCX) [file pone.0265501.s001.docx]

**S1 Table.** Descriptive values (mean ± SD) per position (center back, full back, central midfielder, wide midfielder, forward) depending on the tactical formation.

| **position** | **formation** | **sample** | **Mean** | **SD** | **position** | | **formation** | **sample** | **Mean** | **SD** |  |
| --- | --- | --- | --- | --- | --- | --- | --- | --- | --- | --- | --- |
| **total distance [km]** | | | | | | **dribblings [quantity]** | | | | | |
| CB | 4-4-2 | 32 | 10.14 | 0.65 | CB | | 4-4-2 | 32 | 0.31 | 0.54 |  |
|  | 4-4-2 dia. | 121 | 10.14 | 0.58 |  | | 4-4-2 dia. | 121 | 0.23 | 0.57 |  |
|  | 4-2-2-2 | 89 | 10.05 | 0.49 |  | | 4-2-2-2 | 89 | 0.20 | 0.48 |  |
|  | 4-3-3 | 209 | 10.48 | 0.59 |  | | 4-3-3 | 209 | 0.19 | 0.45 |  |
|  | 4-5-1 | 85 | 10.01 | 0.71 |  | | 4-5-1 | 85 | 0.09 | 0.40 |  |
|  | 4-2-3-1 | 195 | 10.20 | 0.59 |  | | 4-2-3-1 | 195 | 0.19 | 0.46 |  |
|  | 3-4-3 | 212 | 10.33 | 0.68 |  | | 3-4-3 | 212 | 0.23 | 0.56 |  |
|  | 3-5-2 | 184 | 10.21 | 0.67 |  | | 3-5-2 | 184 | 0.22 | 0.54 |  |
| FB | 4-4-2 | 29 | 10.69 | 0.65 | FB | | 4-4-2 | 29 | 0.79 | 0.94 |  |
|  | 4-4-2 dia. | 110 | 10.87 | 0.64 |  | | 4-4-2 dia. | 110 | 1.09 | 1.24 |  |
|  | 4-2-2-2 | 79 | 10.80 | 0.56 |  | | 4-2-2-2 | 79 | 0.90 | 1.10 |  |
|  | 4-3-3 | 183 | 10.98 | 0.68 |  | | 4-3-3 | 183 | 0.77 | 1.11 |  |
|  | 4-5-1 | 82 | 10.67 | 0.65 |  | | 4-5-1 | 82 | 0.80 | 1.06 |  |
|  | 4-2-3-1 | 181 | 10.81 | 0.65 |  | | 4-2-3-1 | 181 | 1.17 | 1.28 |  |
|  | 3-4-3 | 131 | 11.05 | 0.60 |  | | 3-4-3 | 131 | 1.38 | 1.45 |  |
|  | 3-5-2 | 118 | 11.03 | 0.67 |  | | 3-5-2 | 118 | 1.51 | 1.68 |  |
| CM | 4-4-2 | 24 | 11.67 | 0.51 | CM | | 4-4-2 | 24 | 0.33 | 0.64 |  |
|  | 4-4-2 dia. | 83 | 11.32 | 0.67 |  | | 4-4-2 dia. | 83 | 0.94 | 1.18 |  |
|  | 4-2-2-2 | 67 | 11.74 | 0.60 |  | | 4-2-2-2 | 67 | 0.61 | 1.09 |  |
|  | 4-3-3 | 221 | 11.78 | 0.67 |  | | 4-3-3 | 221 | 0.75 | 1.05 |  |
|  | 4-5-1 | 101 | 11.66 | 0.68 |  | | 4-5-1 | 101 | 0.84 | 1.23 |  |
|  | 4-2-3-1 | 210 | 11.64 | 0.71 |  | | 4-2-3-1 | 210 | 0.80 | 1.07 |  |
|  | 3-4-3 | 98 | 11.61 | 0.71 |  | | 3-4-3 | 98 | 0.65 | 0.90 |  |
|  | 3-5-2 | 123 | 11.81 | 0.66 |  | | 3-5-2 | 123 | 1.04 | 1.38 |  |
| WM | 4-4-2 | 11 | 11.50 | 0.60 | WM | | 4-4-2 | 11 | 2.45 | 1.81 |  |
|  | 4-4-2 dia. | 77 | 11.78 | 0.65 |  | | 4-4-2 dia. | 77 | 1.18 | 1.32 |  |
|  | 4-2-2-2 | 44 | 11.17 | 0.58 |  | | 4-2-2-2 | 44 | 2.36 | 2.28 |  |
|  | 4-3-3 | 88 | 11.16 | 0.66 |  | | 4-3-3 | 88 | 2.24 | 1.97 |  |
|  | 4-5-1 | 43 | 11.15 | 0.67 |  | | 4-5-1 | 43 | 2.07 | 1.89 |  |
|  | 4-2-3-1 | 112 | 11.28 | 0.85 |  | | 4-2-3-1 | 112 | 2.40 | 2.22 |  |
|  | 3-4-3 | 85 | 10.70 | 0.91 |  | | 3-4-3 | 85 | 1.47 | 1.74 |  |
| F | 4-4-2 | 18 | 11.20 | 1.13 | F | | 4-4-2 | 18 | 1.17 | 1.69 |  |
|  | 4-4-2 dia. | 57 | 11.00 | 0.65 |  | | 4-4-2 dia. | 57 | 1.75 | 1.98 |  |
|  | 4-2-2-2 | 48 | 11.29 | 0.81 |  | | 4-2-2-2 | 48 | 1.35 | 1.68 |  |
|  | 4-3-3 | 74 | 10.92 | 0.64 |  | | 4-3-3 | 74 | 0.54 | 0.83 |  |
|  | 4-5-1 | 22 | 10.87 | 0.81 |  | | 4-5-1 | 22 | 0.95 | 1.62 |  |
|  | 4-2-3-1 | 54 | 10.97 | 0.94 |  | | 4-2-3-1 | 54 | 1.22 | 1.19 |  |
|  | 3-4-3 | 43 | 10.98 | 0.85 |  | | 3-4-3 | 43 | 1.44 | 1.39 |  |
|  | 3-5-2 | 67 | 10.62 | 0.77 |  | | 3-5-2 | 67 | 1.51 | 1.53 |  |
| **high-intensity distance [km]** | | | | | | **passes short [quantity]** | | | | | |
| CB | 4-4-2 | 32 | 0.96 | 0.27 | CB | | 4-4-2 | 32 | 8.22 | 4.80 |  |
|  | 4-4-2 dia. | 121 | 1.00 | 0.17 |  | | 4-4-2 dia. | 121 | 9.69 | 8.05 |  |
|  | 4-2-2-2 | 89 | 0.88 | 0.17 |  | | 4-2-2-2 | 89 | 10.57 | 8.03 |  |
|  | 4-3-3 | 209 | 1.01 | 0.24 |  | | 4-3-3 | 209 | 13.86 | 10.14 |  |
|  | 4-5-1 | 85 | 0.90 | 0.25 |  | | 4-5-1 | 85 | 10.14 | 6.25 |  |
|  | 4-2-3-1 | 195 | 0.96 | 0.18 |  | | 4-2-3-1 | 195 | 14.77 | 9.37 |  |
|  | 3-4-3 | 212 | 1.08 | 0.22 |  | | 3-4-3 | 212 | 13.57 | 9.33 |  |
|  | 3-5-2 | 184 | 1.05 | 0.24 |  | | 3-5-2 | 184 | 13.26 | 8.63 |  |
| FB | 4-4-2 | 29 | 1.23 | 0.33 | FB | | 4-4-2 | 29 | 8.62 | 4.46 |  |
|  | 4-4-2 dia. | 110 | 1.41 | 0.22 |  | | 4-4-2 dia. | 110 | 13.99 | 7.88 |  |
|  | 4-2-2-2 | 79 | 1.29 | 0.20 |  | | 4-2-2-2 | 79 | 14.63 | 8.66 |  |
|  | 4-3-3 | 183 | 1.43 | 0.26 |  | | 4-3-3 | 183 | 18.11 | 11.71 |  |
|  | 4-5-1 | 82 | 1.26 | 0.29 |  | | 4-5-1 | 82 | 12.65 | 7.62 |  |
|  | 4-2-3-1 | 181 | 1.38 | 0.24 |  | | 4-2-3-1 | 181 | 21.49 | 13.42 |  |
|  | 3-4-3 | 131 | 1.56 | 0.25 |  | | 3-4-3 | 131 | 14.29 | 7.57 |  |
|  | 3-5-2 | 118 | 1.49 | 0.25 |  | | 3-5-2 | 118 | 13.39 | 6.16 |  |
| CM | 4-4-2 | 24 | 1.41 | 0.23 | CM | | 4-4-2 | 24 | 15.42 | 6.57 |  |
|  | 4-4-2 dia. | 83 | 1.48 | 0.30 |  | | 4-4-2 dia. | 83 | 18.17 | 10.69 |  |
|  | 4-2-2-2 | 67 | 1.57 | 0.30 |  | | 4-2-2-2 | 67 | 19.37 | 12.53 |  |
|  | 4-3-3 | 221 | 1.58 | 0.34 |  | | 4-3-3 | 221 | 19.80 | 12.04 |  |
|  | 4-5-1 | 101 | 1.54 | 0.35 |  | | 4-5-1 | 101 | 15.30 | 8.69 |  |
|  | 4-2-3-1 | 210 | 1.58 | 0.30 |  | | 4-2-3-1 | 210 | 23.32 | 12.70 |  |
|  | 3-4-3 | 98 | 1.58 | 0.38 |  | | 3-4-3 | 98 | 20.00 | 9.96 |  |
|  | 3-5-2 | 123 | 1.61 | 0.32 |  | | 3-5-2 | 123 | 16.49 | 7.56 |  |
| WM | 4-4-2 | 11 | 1.62 | 0.22 | WM | | 4-4-2 | 11 | 11.73 | 5.87 |  |
|  | 4-4-2 dia. | 77 | 1.79 | 0.32 |  | | 4-4-2 dia. | 77 | 18.70 | 9.75 |  |
|  | 4-2-2-2 | 44 | 1.48 | 0.22 |  | | 4-2-2-2 | 44 | 14.36 | 7.97 |  |
|  | 4-3-3 | 88 | 1.59 | 0.24 |  | | 4-3-3 | 88 | 16.63 | 11.06 |  |
|  | 4-5-1 | 43 | 1.46 | 0.24 |  | | 4-5-1 | 43 | 11.49 | 8.04 |  |
|  | 4-2-3-1 | 112 | 1.59 | 0.24 |  | | 4-2-3-1 | 112 | 18.11 | 10.93 |  |
|  | 3-4-3 | 85 | 1.42 | 0.33 |  | | 3-4-3 | 85 | 13.96 | 7.64 |  |
| F | 4-4-2 | 18 | 1.41 | 0.39 | F | | 4-4-2 | 18 | 10.29 | 5.73 |  |
|  | 4-4-2 dia. | 57 | 1.51 | 0.33 |  | | 4-4-2 dia. | 57 | 14.01 | 9.38 |  |
|  | 4-2-2-2 | 48 | 1.48 | 0.25 |  | | 4-2-2-2 | 48 | 14.14 | 9.44 |  |
|  | 4-3-3 | 74 | 1.47 | 0.30 |  | | 4-3-3 | 74 | 16.75 | 11.16 |  |
|  | 4-5-1 | 22 | 1.41 | 0.29 |  | | 4-5-1 | 22 | 12.43 | 7.76 |  |
|  | 4-2-3-1 | 54 | 1.42 | 0.38 |  | | 4-2-3-1 | 54 | 19.07 | 12.15 |  |
|  | 3-4-3 | 43 | 1.45 | 0.32 |  | | 3-4-3 | 43 | 15.09 | 8.95 |  |
|  | 3-5-2 | 67 | 1.45 | 0.31 |  | | 3-5-2 | 67 | 13.93 | 7.54 |  |
| **sprinting distance [km]** | | | | | | **passes middle [quantity]** | | | | | |
| CB | 4-4-2 | 32 | 0.16 | 0.08 | CB | | 4-4-2 | 32 | 33.47 | 14.33 |  |
|  | 4-4-2 dia. | 121 | 0.18 | 0.09 |  | | 4-4-2 dia. | 121 | 30.40 | 15.24 |  |
|  | 4-2-2-2 | 89 | 0.16 | 0.09 |  | | 4-2-2-2 | 89 | 29.94 | 16.61 |  |
|  | 4-3-3 | 209 | 0.16 | 0.08 |  | | 4-3-3 | 209 | 45.95 | 21.05 |  |
|  | 4-5-1 | 85 | 0.17 | 0.08 |  | | 4-5-1 | 85 | 29.28 | 16.20 |  |
|  | 4-2-3-1 | 195 | 0.17 | 0.07 |  | | 4-2-3-1 | 195 | 38.67 | 18.20 |  |
|  | 3-4-3 | 212 | 0.22 | 0.09 |  | | 3-4-3 | 212 | 29.21 | 15.09 |  |
|  | 3-5-2 | 184 | 0.22 | 0.10 |  | | 3-5-2 | 184 | 24.67 | 13.58 |  |
| FB | 4-4-2 | 29 | 0.26 | 0.11 | FB | | 4-4-2 | 29 | 17.90 | 6.07 |  |
|  | 4-4-2 dia. | 110 | 0.37 | 0.13 |  | | 4-4-2 dia. | 110 | 19.70 | 9.10 |  |
|  | 4-2-2-2 | 79 | 0.32 | 0.12 |  | | 4-2-2-2 | 79 | 19.57 | 8.38 |  |
|  | 4-3-3 | 183 | 0.35 | 0.11 |  | | 4-3-3 | 183 | 25.14 | 11.49 |  |
|  | 4-5-1 | 82 | 0.32 | 0.11 |  | | 4-5-1 | 82 | 17.65 | 7.03 |  |
|  | 4-2-3-1 | 181 | 0.37 | 0.14 |  | | 4-2-3-1 | 181 | 22.85 | 10.58 |  |
|  | 3-4-3 | 131 | 0.40 | 0.13 |  | | 3-4-3 | 131 | 16.76 | 7.85 |  |
|  | 3-5-2 | 118 | 0.39 | 0.13 |  | | 3-5-2 | 118 | 15.69 | 6.80 |  |
| CM | 4-4-2 | 24 | 0.21 | 0.09 | CM | | 4-4-2 | 24 | 24.25 | 9.62 |  |
|  | 4-4-2 dia. | 83 | 0.27 | 0.13 |  | | 4-4-2 dia. | 83 | 21.12 | 9.33 |  |
|  | 4-2-2-2 | 67 | 0.23 | 0.11 |  | | 4-2-2-2 | 67 | 24.34 | 11.43 |  |
|  | 4-3-3 | 221 | 0.26 | 0.13 |  | | 4-3-3 | 221 | 25.10 | 13.10 |  |
|  | 4-5-1 | 101 | 0.24 | 0.11 |  | | 4-5-1 | 101 | 18.65 | 8.66 |  |
|  | 4-2-3-1 | 210 | 0.26 | 0.13 |  | | 4-2-3-1 | 210 | 24.13 | 12.95 |  |
|  | 3-4-3 | 98 | 0.25 | 0.10 |  | | 3-4-3 | 98 | 21.10 | 9.31 |  |
|  | 3-5-2 | 123 | 0.27 | 0.12 |  | | 3-5-2 | 123 | 17.73 | 8.41 |  |
| WM | 4-4-2 | 11 | 0.40 | 0.12 | WM | | 4-4-2 | 11 | 14.91 | 3.65 |  |
|  | 4-4-2 dia. | 77 | 0.33 | 0.12 |  | | 4-4-2 dia. | 77 | 17.78 | 7.83 |  |
|  | 4-2-2-2 | 44 | 0.36 | 0.12 |  | | 4-2-2-2 | 44 | 12.64 | 6.68 |  |
|  | 4-3-3 | 88 | 0.41 | 0.14 |  | | 4-3-3 | 88 | 14.47 | 6.80 |  |
|  | 4-5-1 | 43 | 0.47 | 0.18 |  | | 4-5-1 | 43 | 10.30 | 4.45 |  |
|  | 4-2-3-1 | 112 | 0.43 | 0.17 |  | | 4-2-3-1 | 112 | 14.29 | 6.53 |  |
|  | 3-4-3 | 85 | 0.35 | 0.12 |  | | 3-4-3 | 85 | 9.05 | 6.17 |  |
| F | 4-4-2 | 18 | 0.32 | 0.15 | F | | 4-4-2 | 18 | 10.22 | 4.25 |  |
|  | 4-4-2 dia. | 57 | 0.43 | 0.11 |  | | 4-4-2 dia. | 57 | 8.28 | 5.00 |  |
|  | 4-2-2-2 | 48 | 0.32 | 0.11 |  | | 4-2-2-2 | 48 | 9.25 | 6.33 |  |
|  | 4-3-3 | 74 | 0.31 | 0.13 |  | | 4-3-3 | 74 | 10.58 | 7.65 |  |
|  | 4-5-1 | 22 | 0.32 | 0.13 |  | | 4-5-1 | 22 | 6.05 | 2.84 |  |
|  | 4-2-3-1 | 54 | 0.32 | 0.11 |  | | 4-2-3-1 | 54 | 8.15 | 3.70 |  |
|  | 3-4-3 | 43 | 0.34 | 0.14 |  | | 3-4-3 | 43 | 11.26 | 7.99 |  |
|  | 3-5-2 | 67 | 0.36 | 0.12 |  | | 3-5-2 | 67 | 7.96 | 3.70 |  |
| **max. velocity [km/h]** | | | | | | **passes long [quantity]** | | | | | |
| CB | 4-4-2 | 32 | 30.80 | 1.20 | CB | | 4-4-2 | 32 | 5.53 | 2.96 |  |
|  | 4-4-2 dia. | 121 | 30.76 | 1.67 |  | | 4-4-2 dia. | 121 | 6.13 | 4.42 |  |
|  | 4-2-2-2 | 89 | 30.17 | 1.75 |  | | 4-2-2-2 | 89 | 5.54 | 3.66 |  |
|  | 4-3-3 | 209 | 30.37 | 1.85 |  | | 4-3-3 | 209 | 6.02 | 4.39 |  |
|  | 4-5-1 | 85 | 30.68 | 1.63 |  | | 4-5-1 | 85 | 5.55 | 3.36 |  |
|  | 4-2-3-1 | 195 | 30.75 | 2.18 |  | | 4-2-3-1 | 195 | 5.36 | 3.69 |  |
|  | 3-4-3 | 212 | 30.91 | 1.56 |  | | 3-4-3 | 212 | 5.46 | 3.77 |  |
|  | 3-5-2 | 184 | 30.97 | 1.67 |  | | 3-5-2 | 184 | 4.77 | 3.19 |  |
| FB | 4-4-2 | 29 | 31.03 | 2.07 | FB | | 4-4-2 | 29 | 4.14 | 2.66 |  |
|  | 4-4-2 dia. | 110 | 31.44 | 1.45 |  | | 4-4-2 dia. | 110 | 3.67 | 2.54 |  |
|  | 4-2-2-2 | 79 | 31.63 | 1.75 |  | | 4-2-2-2 | 79 | 3.70 | 2.75 |  |
|  | 4-3-3 | 183 | 31.35 | 1.29 |  | | 4-3-3 | 183 | 3.24 | 2.53 |  |
|  | 4-5-1 | 82 | 31.39 | 1.26 |  | | 4-5-1 | 82 | 3.87 | 2.56 |  |
|  | 4-2-3-1 | 181 | 31.86 | 1.56 |  | | 4-2-3-1 | 181 | 3.06 | 2.65 |  |
|  | 3-4-3 | 131 | 31.72 | 1.45 |  | | 3-4-3 | 131 | 3.08 | 2.28 |  |
|  | 3-5-2 | 118 | 31.58 | 1.52 |  | | 3-5-2 | 118 | 2.66 | 2.30 |  |
| CM | 4-4-2 | 24 | 30.20 | 1.41 | CM | | 4-4-2 | 24 | 2.08 | 1.59 |  |
|  | 4-4-2 dia. | 83 | 30.89 | 1.72 |  | | 4-4-2 dia. | 83 | 3.35 | 3.21 |  |
|  | 4-2-2-2 | 67 | 30.19 | 1.43 |  | | 4-2-2-2 | 67 | 3.28 | 2.71 |  |
|  | 4-3-3 | 221 | 30.52 | 1.68 |  | | 4-3-3 | 221 | 3.19 | 2.78 |  |
|  | 4-5-1 | 101 | 30.23 | 1.35 |  | | 4-5-1 | 101 | 2.55 | 2.11 |  |
|  | 4-2-3-1 | 210 | 30.39 | 1.64 |  | | 4-2-3-1 | 210 | 2.67 | 2.74 |  |
|  | 3-4-3 | 98 | 30.25 | 1.54 |  | | 3-4-3 | 98 | 2.22 | 1.80 |  |
|  | 3-5-2 | 123 | 30.41 | 2.29 |  | | 3-5-2 | 123 | 2.37 | 2.11 |  |
| WM | 4-4-2 | 11 | 31.79 | 1.37 | WM | | 4-4-2 | 11 | 1.36 | 1.36 |  |
|  | 4-4-2 dia. | 77 | 30.66 | 2.70 |  | | 4-4-2 dia. | 77 | 2.56 | 2.16 |  |
|  | 4-2-2-2 | 44 | 31.70 | 1.30 |  | | 4-2-2-2 | 44 | 1.68 | 1.88 |  |
|  | 4-3-3 | 88 | 31.85 | 1.44 |  | | 4-3-3 | 88 | 1.35 | 1.47 |  |
|  | 4-5-1 | 43 | 32.10 | 1.04 |  | | 4-5-1 | 43 | 1.26 | 1.38 |  |
|  | 4-2-3-1 | 112 | 31.97 | 1.51 |  | | 4-2-3-1 | 112 | 1.33 | 1.42 |  |
|  | 3-4-3 | 85 | 31.31 | 1.25 |  | | 3-4-3 | 85 | 1.06 | 1.37 |  |
| F | 4-4-2 | 18 | 30.87 | 1.41 | F | | 4-4-2 | 18 | 0.56 | 0.86 |  |
|  | 4-4-2 dia. | 57 | 32.20 | 1.15 |  | | 4-4-2 dia. | 57 | 0.53 | 0.85 |  |
|  | 4-2-2-2 | 48 | 30.88 | 1.76 |  | | 4-2-2-2 | 48 | 0.75 | 1.49 |  |
|  | 4-3-3 | 74 | 30.92 | 1.37 |  | | 4-3-3 | 74 | 1.01 | 1.65 |  |
|  | 4-5-1 | 22 | 31.40 | 1.71 |  | | 4-5-1 | 22 | 0.36 | 0.73 |  |
|  | 4-2-3-1 | 54 | 31.08 | 1.31 |  | | 4-2-3-1 | 54 | 0.50 | 0.75 |  |
|  | 3-4-3 | 43 | 31.32 | 1.39 |  | | 3-4-3 | 43 | 1.09 | 1.25 |  |
|  | 3-5-2 | 67 | 31.65 | 1.55 |  | | 3-5-2 | 67 | 0.57 | 0.82 |  |
| **accelerations [quantity]** | | | | | | **ball-possession phases [quantity]** | | | | | |
| CB | 4-4-2 | 32 | 481.13 | 39.12 | CB | | 4-4-2 | 32 | 64.81 | 18.48 |  |
|  | 4-4-2 dia. | 121 | 471.07 | 34.78 |  | | 4-4-2 dia. | 121 | 63.16 | 19.59 |  |
|  | 4-2-2-2 | 89 | 471.78 | 32.14 |  | | 4-2-2-2 | 89 | 62.30 | 21.20 |  |
|  | 4-3-3 | 205 | 489.76 | 35.77 |  | | 4-3-3 | 209 | 80.54 | 27.28 |  |
|  | 4-5-1 | 85 | 480.66 | 38.31 |  | | 4-5-1 | 85 | 60.91 | 20.15 |  |
|  | 4-2-3-1 | 190 | 482.94 | 37.26 |  | | 4-2-3-1 | 195 | 73.88 | 24.21 |  |
|  | 3-4-3 | 212 | 478.80 | 35.26 |  | | 3-4-3 | 212 | 64.45 | 19.75 |  |
|  | 3-5-2 | 176 | 474.84 | 36.46 |  | | 3-5-2 | 184 | 59.86 | 18.24 |  |
| FB | 4-4-2 | 29 | 507.83 | 34.56 | FB | | 4-4-2 | 29 | 58.62 | 13.44 |  |
|  | 4-4-2 dia. | 110 | 492.22 | 37.03 |  | | 4-4-2 dia. | 110 | 65.67 | 15.83 |  |
|  | 4-2-2-2 | 79 | 503.23 | 38.15 |  | | 4-2-2-2 | 79 | 65.48 | 16.82 |  |
|  | 4-3-3 | 180 | 510.58 | 38.29 |  | | 4-3-3 | 183 | 72.81 | 19.49 |  |
|  | 4-5-1 | 82 | 499.50 | 39.93 |  | | 4-5-1 | 82 | 59.44 | 14.06 |  |
|  | 4-2-3-1 | 175 | 505.46 | 37.49 |  | | 4-2-3-1 | 181 | 74.67 | 19.39 |  |
|  | 3-4-3 | 131 | 501.37 | 34.46 |  | | 3-4-3 | 131 | 62.49 | 14.60 |  |
|  | 3-5-2 | 112 | 504.22 | 33.75 |  | | 3-5-2 | 118 | 58.69 | 13.33 |  |
| CM | 4-4-2 | 24 | 533.71 | 25.53 | CM | | 4-4-2 | 24 | 57.38 | 13.41 |  |
|  | 4-4-2 dia. | 83 | 497.87 | 36.03 |  | | 4-4-2 dia. | 83 | 61.58 | 16.94 |  |
|  | 4-2-2-2 | 67 | 522.12 | 28.93 |  | | 4-2-2-2 | 67 | 64.85 | 20.75 |  |
|  | 4-3-3 | 217 | 515.96 | 42.49 |  | | 4-3-3 | 221 | 64.18 | 22.84 |  |
|  | 4-5-1 | 101 | 512.51 | 38.93 |  | | 4-5-1 | 101 | 52.68 | 15.74 |  |
|  | 4-2-3-1 | 206 | 511.36 | 40.43 |  | | 4-2-3-1 | 210 | 66.23 | 21.72 |  |
|  | 3-4-3 | 98 | 526.88 | 41.93 |  | | 3-4-3 | 98 | 60.94 | 16.35 |  |
|  | 3-5-2 | 119 | 514.52 | 37.65 |  | | 3-5-2 | 123 | 54.74 | 14.27 |  |
| WM | 4-4-2 | 11 | 516.82 | 50.88 | WM | | 4-4-2 | 11 | 48.36 | 9.27 |  |
|  | 4-4-2 dia. | 77 | 512.94 | 32.86 |  | | 4-4-2 dia. | 77 | 56.51 | 14.52 |  |
|  | 4-2-2-2 | 44 | 499.61 | 36.72 |  | | 4-2-2-2 | 44 | 47.59 | 13.94 |  |
|  | 4-3-3 | 86 | 485.56 | 37.63 |  | | 4-3-3 | 88 | 51.91 | 17.97 |  |
|  | 4-5-1 | 43 | 483.44 | 36.64 |  | | 4-5-1 | 43 | 42.00 | 14.06 |  |
|  | 4-2-3-1 | 109 | 504.14 | 40.59 |  | | 4-2-3-1 | 112 | 53.32 | 15.98 |  |
|  | 3-4-3 | 85 | 463.78 | 45.92 |  | | 3-4-3 | 85 | 42.35 | 13.05 |  |
| F | 4-4-2 | 18 | 486.56 | 47.29 | F | | 4-4-2 | 18 | 35.56 | 8.37 |  |
|  | 4-4-2 dia. | 57 | 466.16 | 40.54 |  | | 4-4-2 dia. | 57 | 37.75 | 8.60 |  |
|  | 4-2-2-2 | 48 | 480.79 | 36.78 |  | | 4-2-2-2 | 48 | 38.81 | 12.23 |  |
|  | 4-3-3 | 72 | 478.85 | 32.94 |  | | 4-3-3 | 74 | 40.70 | 14.58 |  |
|  | 4-5-1 | 22 | 466.27 | 37.66 |  | | 4-5-1 | 22 | 31.32 | 7.29 |  |
|  | 4-2-3-1 | 51 | 478.65 | 44.76 |  | | 4-2-3-1 | 54 | 36.83 | 11.04 |  |
|  | 3-4-3 | 43 | 477.09 | 40.56 |  | | 3-4-3 | 43 | 46.09 | 12.93 |  |
|  | 3-5-2 | 64 | 459.13 | 43.17 |  | | 3-5-2 | 67 | 37.88 | 9.55 |  |

[dia. = diamond; CB = Center Back; FB = Full Back; CM = Central Midfielder; WM = Wide Midfielder; F = Forward]
