# Supplementary material for: Center backs work hardest when playing in a back three: The influence of tactical formation on physical and technical match performance in professional soccer
Source: PLoS One. 2022 Mar 17;17(3):e0265501. doi: 10.1371/journal.pone.0265501 (PMC8929644; doi:10.1371/journal.pone.0265501)
Supplement: S2 Table — (DOCX) [file pone.0265501.s002.docx]

**S2 Table.** Descriptive values (mean ± SD) depending on the tactical formation.

| **formation** | **sample** | **Mean** | **SD** | **Formation** | **Stichprobe** | **Mean** | **SD** |
| --- | --- | --- | --- | --- | --- | --- | --- |
| **total distance [km]** | | | | **dribblings [quantity]** | | | |
| 4-4-2 | 114 | 10.9 | 0.92 | 4-4-2 | 114 | 0.78 | 1.22 |
| 4-4-2 dia. | 448 | 10.93 | 0.85 | 4-4-2 dia. | 448 | 0.93 | 1.32 |
| 4-2-2-2 | 327 | 10.91 | 0.86 | 4-2-2-2 | 327 | 0.91 | 1.47 |
| 4-3-3 | 775 | 11.09 | 0.81 | 4-3-3 | 775 | 0.75 | 1.22 |
| 4-5-1 | 333 | 10.88 | 0.93 | 4-5-1 | 333 | 0.81 | 1.31 |
| 4-2-3-1 | 752 | 10.96 | 0.9 | 4-2-3-1 | 752 | 1.00 | 1.44 |
| 3-4-3 | 569 | 10.82 | 0.85 | 3-4-3 | 569 | 0.85 | 1.27 |
| 3-5-2 | 492 | 10.86 | 0.93 | 3-5-2 | 492 | 0.91 | 1.38 |
| **high-intensity distance [km]** | | | | **passes short [quantity]** | | | |
| 4-4-2 | 114 | 1.26 | 0.36 | 4-4-2 | 114 | 10.29 | 5.73 |
| 4-4-2 dia. | 448 | 1.39 | 0.37 | 4-4-2 dia. | 448 | 14.01 | 9.38 |
| 4-2-2-2 | 327 | 1.29 | 0.35 | 4-2-2-2 | 327 | 14.14 | 9.44 |
| 4-3-3 | 775 | 1.38 | 0.37 | 4-3-3 | 775 | 16.75 | 11.16 |
| 4-5-1 | 333 | 1.29 | 0.38 | 4-5-1 | 333 | 12.43 | 7.76 |
| 4-2-3-1 | 752 | 1.36 | 0.36 | 4-2-3-1 | 752 | 19.07 | 12.15 |
| 3-4-3 | 569 | 1.36 | 0.36 | 3-4-3 | 569 | 15.09 | 8.95 |
| 3-5-2 | 492 | 1.35 | 0.36 | 3-5-2 | 492 | 13.93 | 7.54 |
| **sprinting distance [km]** | | | | **passes middle [quantity]** | | | |
| 4-4-2 | 114 | 0.25 | 0.13 | 4-4-2 | 114 | 22.11 | 12.56 |
| 4-4-2 dia. | 448 | 0.30 | 0.14 | 4-4-2 dia. | 448 | 21.07 | 12.60 |
| 4-2-2-2 | 327 | 0.26 | 0.13 | 4-2-2-2 | 327 | 20.92 | 13.56 |
| 4-3-3 | 775 | 0.28 | 0.14 | 4-3-3 | 775 | 28.14 | 18.73 |
| 4-5-1 | 333 | 0.28 | 0.15 | 4-5-1 | 333 | 19.21 | 12.35 |
| 4-2-3-1 | 752 | 0.29 | 0.15 | 4-2-3-1 | 752 | 24.98 | 15.93 |
| 3-4-3 | 569 | 0.29 | 0.13 | 3-4-3 | 569 | 20.58 | 13.47 |
| 3-5-2 | 492 | 0.29 | 0.13 | 3-5-2 | 492 | 18.51 | 11.43 |
| **max. velocity [km/h]** | | | | **passes long [quantity]** | | | |
| 4-4-2 | 114 | 30.84 | 1.58 | 4-4-2 | 114 | 3.26 | 2.90 |
| 4-4-2 dia. | 448 | 31.11 | 1.86 | 4-4-2 dia. | 448 | 3.69 | 3.57 |
| 4-2-2-2 | 327 | 30.84 | 1.76 | 4-2-2-2 | 327 | 3.41 | 3.22 |
| 4-3-3 | 775 | 30.86 | 1.67 | 4-3-3 | 775 | 3.55 | 3.50 |
| 4-5-1 | 333 | 30.95 | 1.53 | 4-5-1 | 333 | 3.33 | 2.96 |
| 4-2-3-1 | 752 | 31.12 | 1.85 | 4-2-3-1 | 752 | 3.11 | 3.15 |
| 3-4-3 | 569 | 31.07 | 1.55 | 3-4-3 | 569 | 3.37 | 3.24 |
| 3-5-2 | 492 | 31.07 | 1.85 | 3-5-2 | 492 | 3.09 | 2.89 |
| **accelerations [quantity]** | | | | **ball-possession phases [quantity]** | | | |
| 4-4-2 | 114 | 503.29 | 42.63 | 4-4-2 | 114 | 55.46 | 17.01 |
| 4-4-2 dia. | 448 | 487.80 | 39.43 | 4-4-2 dia. | 448 | 59.11 | 18.37 |
| 4-2-2-2 | 327 | 494.76 | 38.95 | 4-2-2-2 | 327 | 58.17 | 20.50 |
| 4-3-3 | 760 | 500.66 | 40.70 | 4-3-3 | 775 | 66.99 | 25.41 |
| 4-5-1 | 333 | 494.37 | 41.22 | 4-5-1 | 333 | 53.65 | 18.07 |
| 4-2-3-1 | 731 | 499.20 | 41.08 | 4-2-3-1 | 752 | 66.21 | 23.21 |
| 3-4-3 | 569 | 489.91 | 43.52 | 3-4-3 | 569 | 58.70 | 18.58 |
| 3-5-2 | 471 | 489.72 | 42.26 | 3-5-2 | 492 | 55.31 | 16.77 |

[dia. = diamond]
