# Supplementary material for: Center backs work hardest when playing in a back three: The influence of tactical formation on physical and technical match performance in professional soccer
Source: PLoS One. 2022 Mar 17;17(3):e0265501. doi: 10.1371/journal.pone.0265501 (PMC8929644; doi:10.1371/journal.pone.0265501)
Supplement: S3 Table — (DOCX) [file pone.0265501.s003.docx]

**S3 Table.** Descriptive values (mean ± SD) depending on the playing position.

| **position** | **sample** | **Mean** | **SD** | **position** | **sample** | **Mean** | **SD** |
| --- | --- | --- | --- | --- | --- | --- | --- |
| **total distance [km]** | | | | **dribblings [quantity]** | | | |
| CB | 1127 | 10.24 | 0.64 | CB | 1127 | 0.20 | 0.50 |
| FB | 913 | 10.90 | 0.65 | FB | 913 | 1.09 | 1.31 |
| CM | 927 | 11.67 | 0.69 | CM | 927 | 0.80 | 1.12 |
| WM | 460 | 11.21 | 0.82 | WM | 460 | 1.96 | 1.97 |
| F | 383 | 10.95 | 0.81 | F | 383 | 1.24 | 1.51 |
| **high-intensity distance [km]** | | | | **passes short [quantity]** | | | |
| CB | 1127 | 1.00 | 0.22 | CB | 1127 | 12.72 | 9.04 |
| FB | 913 | 1.41 | 0.27 | FB | 913 | 16.03 | 10.35 |
| CM | 927 | 1.57 | 0.33 | CM | 927 | 19.40 | 11.24 |
| WM | 460 | 1.57 | 0.29 | WM | 460 | 16.03 | 9.84 |
| F | 383 | 1.46 | 0.32 | F | 383 | 12.14 | 6.09 |
| **Sprinting distance [km]** | | | | **passes middle [quantity]** | | | |
| CB | 1127 | 0.19 | 0.09 | CB | 1127 | 33.52 | 18.33 |
| FB | 913 | 0.36 | 0.13 | FB | 913 | 20.22 | 9.80 |
| CM | 927 | 0.25 | 0.12 | CM | 927 | 22.35 | 11.49 |
| WM | 460 | 0.39 | 0.15 | WM | 460 | 13.42 | 7.12 |
| F | 383 | 0.34 | 0.13 | F | 383 | 9.07 | 5.86 |
| **max. velocity [km/h]** | | | | **passes long [quantity]** | | | |
| CB | 1127 | 30.69 | 1.79 | CB | 1127 | 5.52 | 3.82 |
| FB | 913 | 31.56 | 1.49 | FB | 913 | 3.28 | 2.54 |
| CM | 927 | 30.42 | 1.70 | CM | 927 | 2.79 | 2.57 |
| WM | 460 | 31.59 | 1.72 | WM | 460 | 1.52 | 1.67 |
| F | 383 | 31.33 | 1.50 | F | 383 | 0.71 | 1.18 |
| **accelerations [quantity]** | | | | **ball-possession phases [quantity]** | | | |
| CB | 1110 | 479.71 | 36.37 | CB | 1127 | 67.75 | 23.21 |
| FB | 898 | 503.45 | 37.17 | FB | 913 | 66.73 | 17.85 |
| CM | 915 | 514.80 | 39.76 | CM | 927 | 61.44 | 19.88 |
| WM | 455 | 492.49 | 42.79 | WM | 460 | 49.83 | 15.97 |
| F | 375 | 473.21 | 40.52 | F | 383 | 38.81 | 11.76 |

[CB = Center Back; FB = Full Back; CM = Central Midfielder; WM = Wide Midfielder; F = Forward]
