# Supplementary material for: Center backs work hardest when playing in a back three: The influence of tactical formation on physical and technical match performance in professional soccer
Source: PLoS One. 2022 Mar 17;17(3):e0265501. doi: 10.1371/journal.pone.0265501 (PMC8929644; doi:10.1371/journal.pone.0265501)
Supplement: S4 Table — (DOCX) [file pone.0265501.s004.docx]

**S4 Table.** Number of players per position (center back, full back, central midfielder, wide midfielder, forward) depending on the tactical formation.

|  | **position** | | | | | |
| --- | --- | --- | --- | --- | --- | --- |
| **formation** |  | center back | full back | central midfielder | Wide midfielder | forward |
|  | 4-4-2 | 2 | 2 | 2 | 2 | 2 |
|  | 4-4-2 dia. | 2 | 2 | 2 | 2 | 2 |
|  | 4-2-2-2 | 2 | 2 | 2 | 2 | 2 |
|  | 4-3-3 | 2 | 2 | 3 | 2 | 1 |
|  | 4-5-1 | 2 | 2 | 3 | 2 | 1 |
|  | 4-2-3-1 | 2 | 2 | 3 | 2 | 1 |
|  | 3-4-3 | 3 | 2 | 2 | 2 | 1 |
|  | 3-5-2 | 3 | 2 | 3 | 0 | 2 |
|  | 3-4-3 dia. | 3 | 2 | 2 | 2 | 1 |

dia. = diamond
