# Supplementary figures and images for: Center backs work hardest when playing in a back three: The influence of tactical formation on physical and technical match performance in professional soccer
Source: PLoS One. 2022 Mar 17;17(3):e0265501. doi: 10.1371/journal.pone.0265501 (PMC8929644; doi:10.1371/journal.pone.0265501)

## 4-4-2

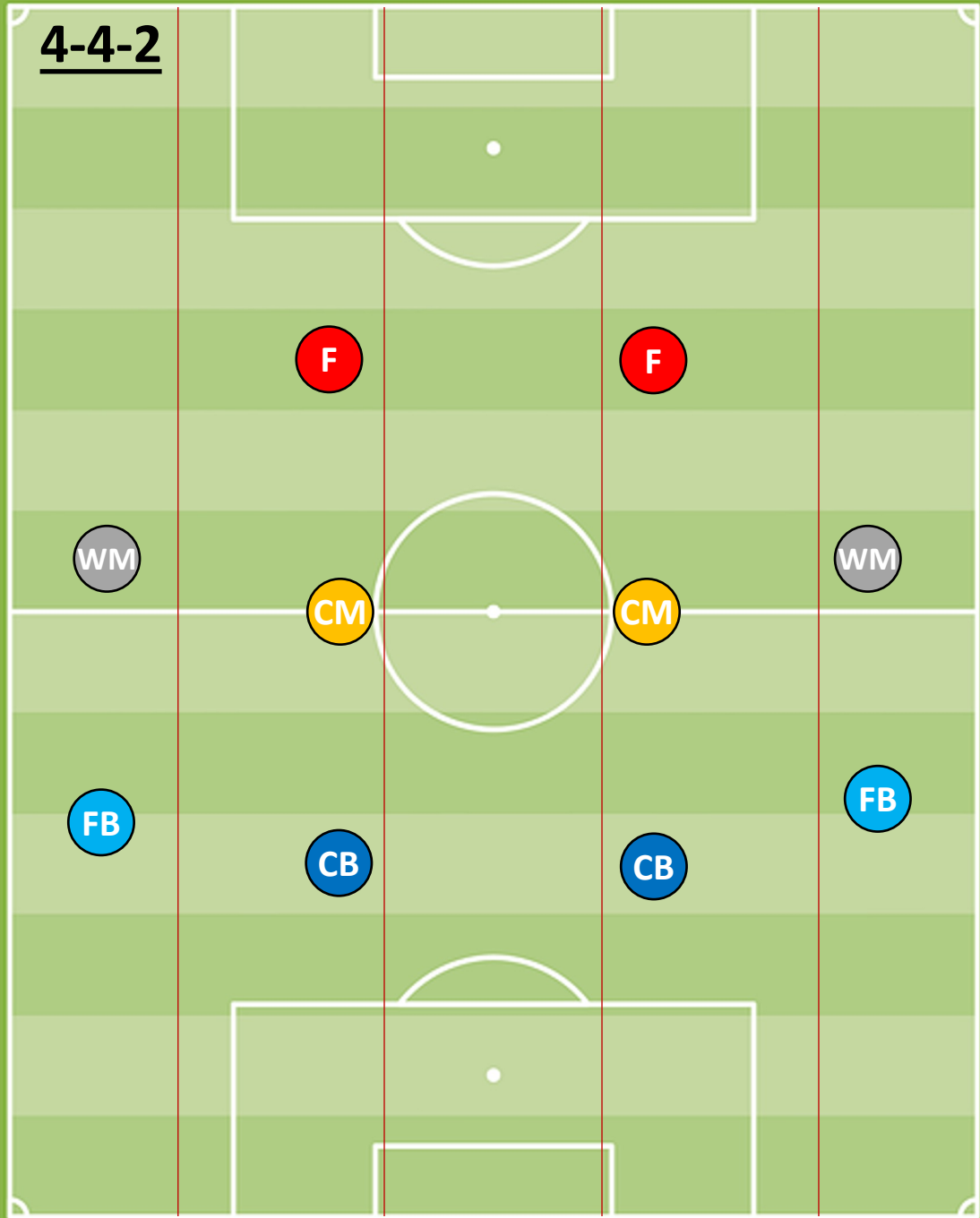

## 4-4-2 diamond

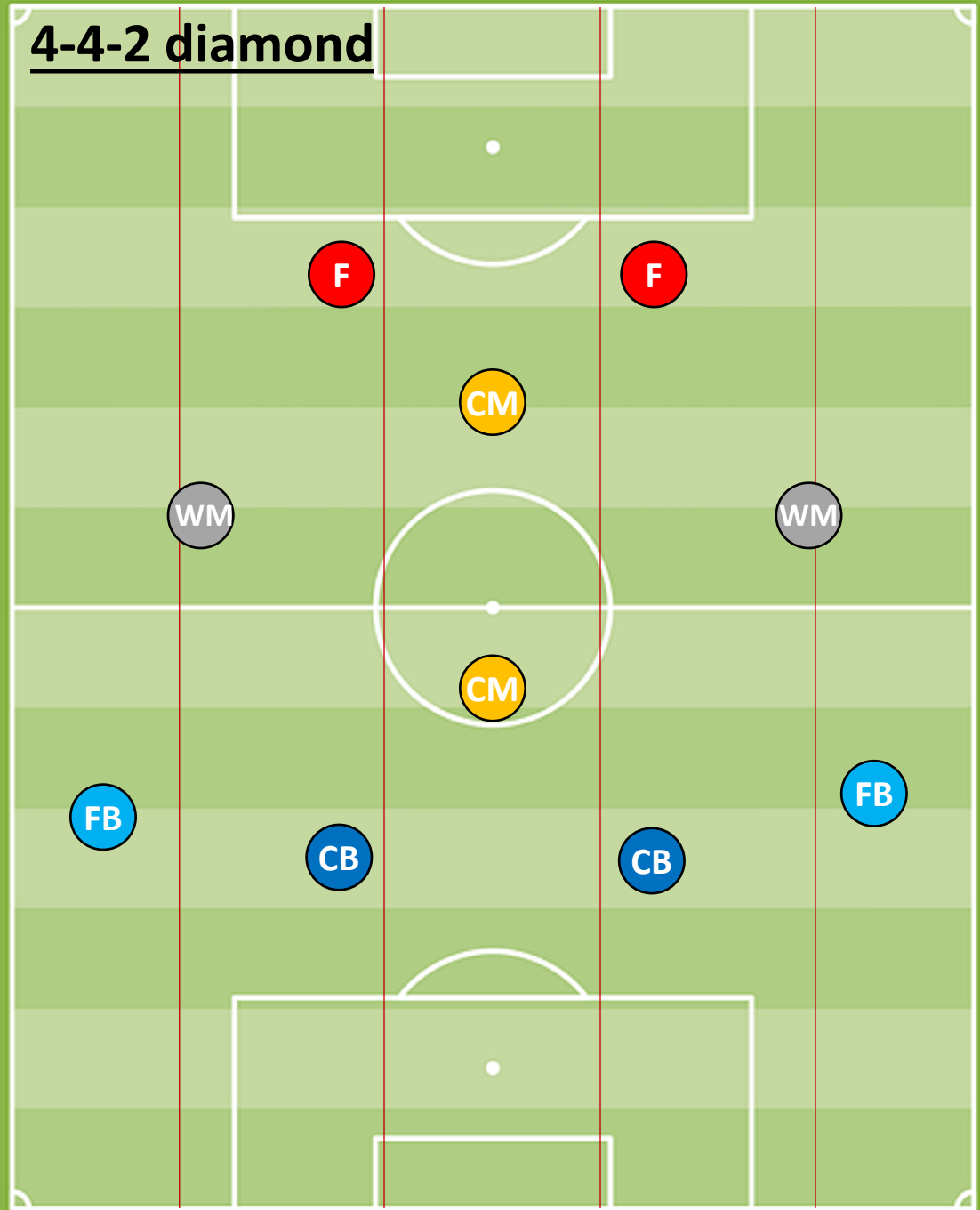

## 4-2-2-2

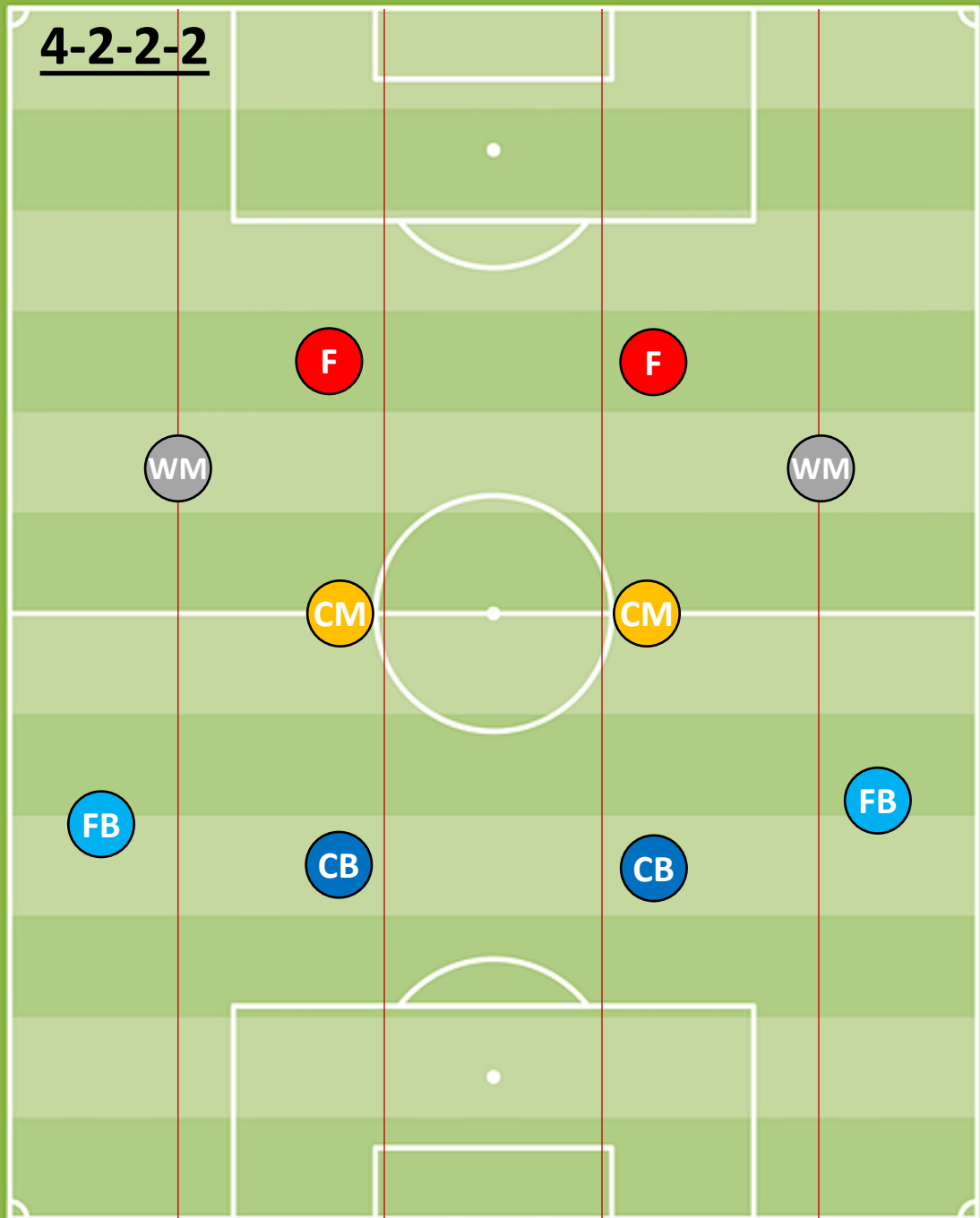

## 4-3-3

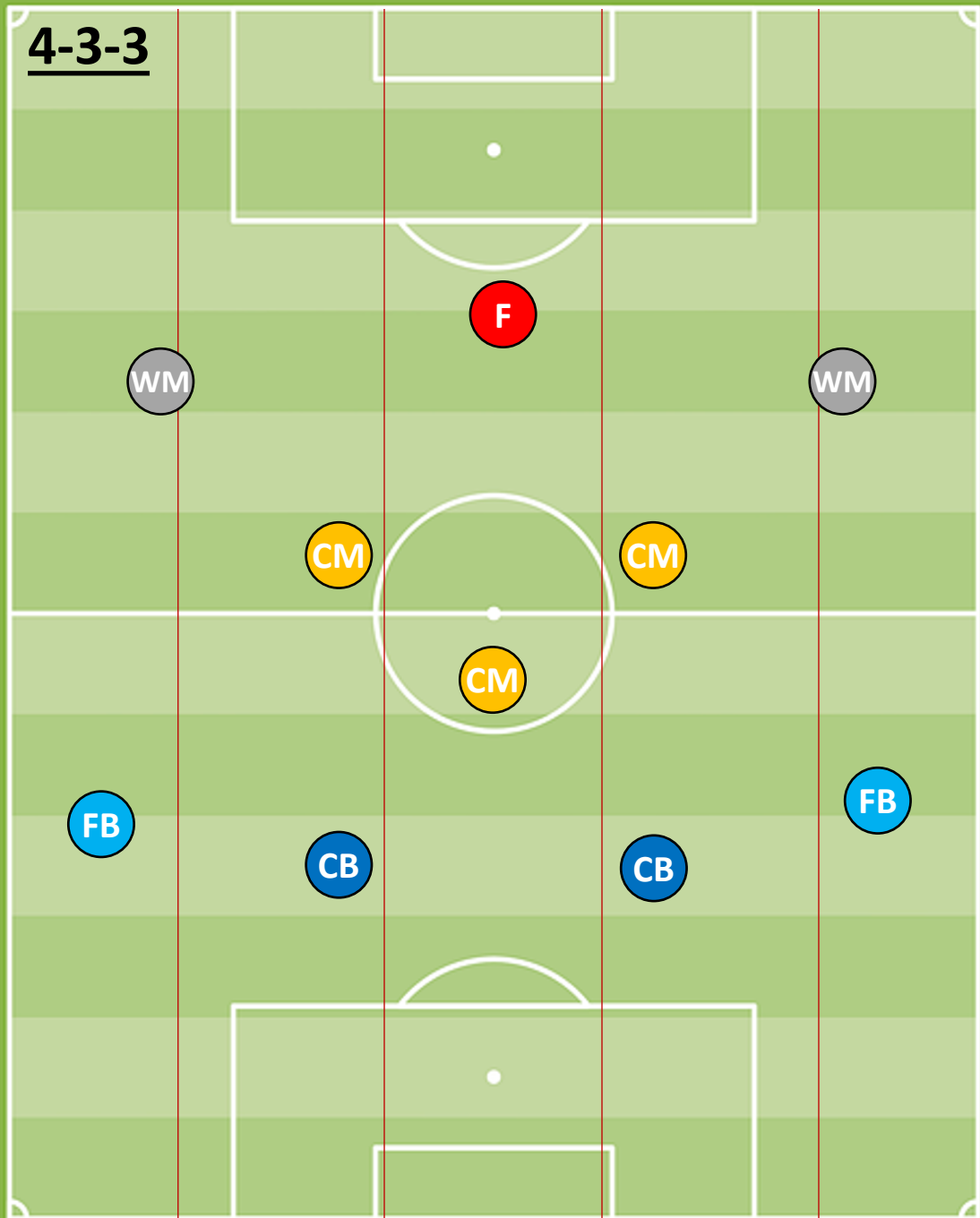

4-5-1

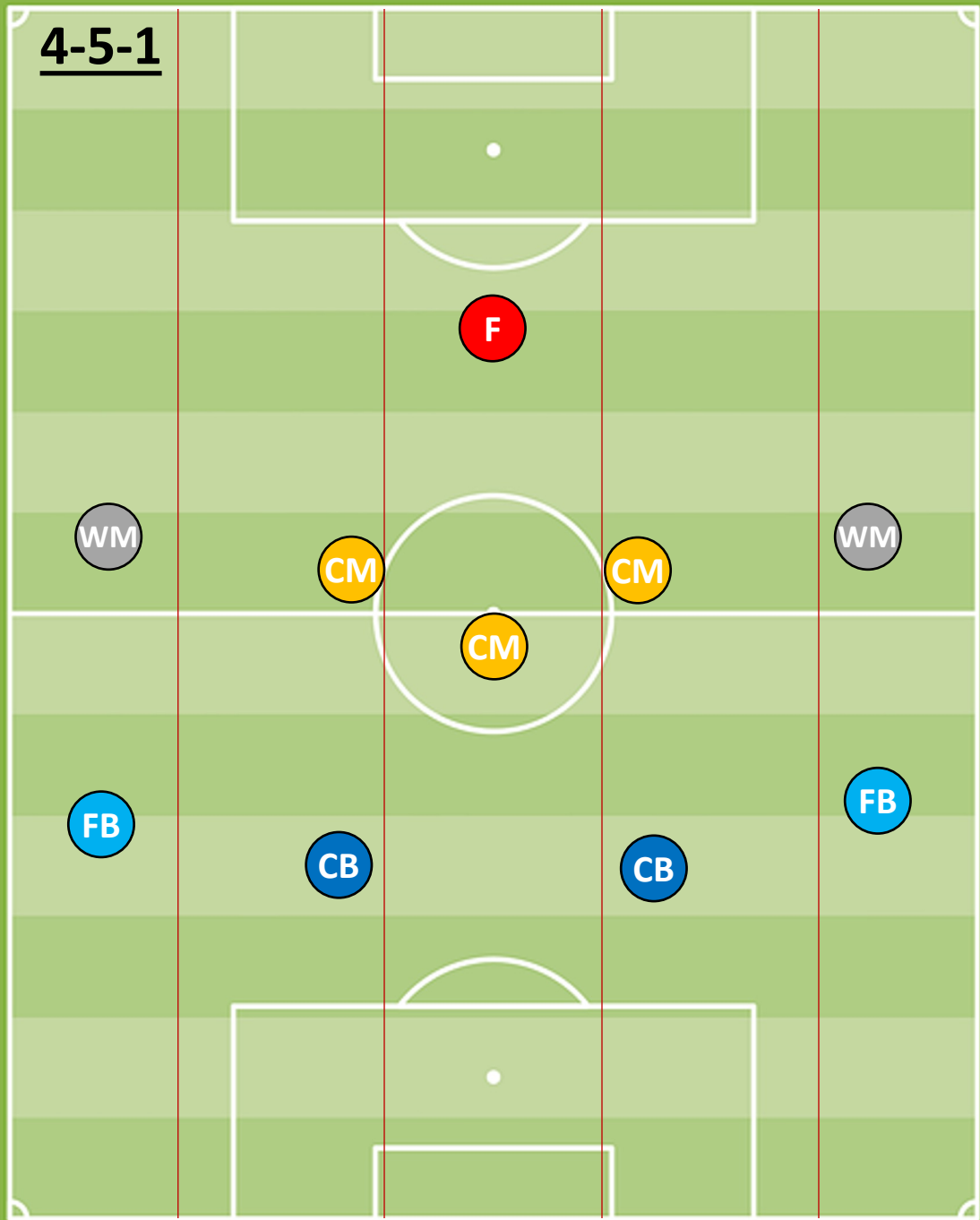

4-2-3-1

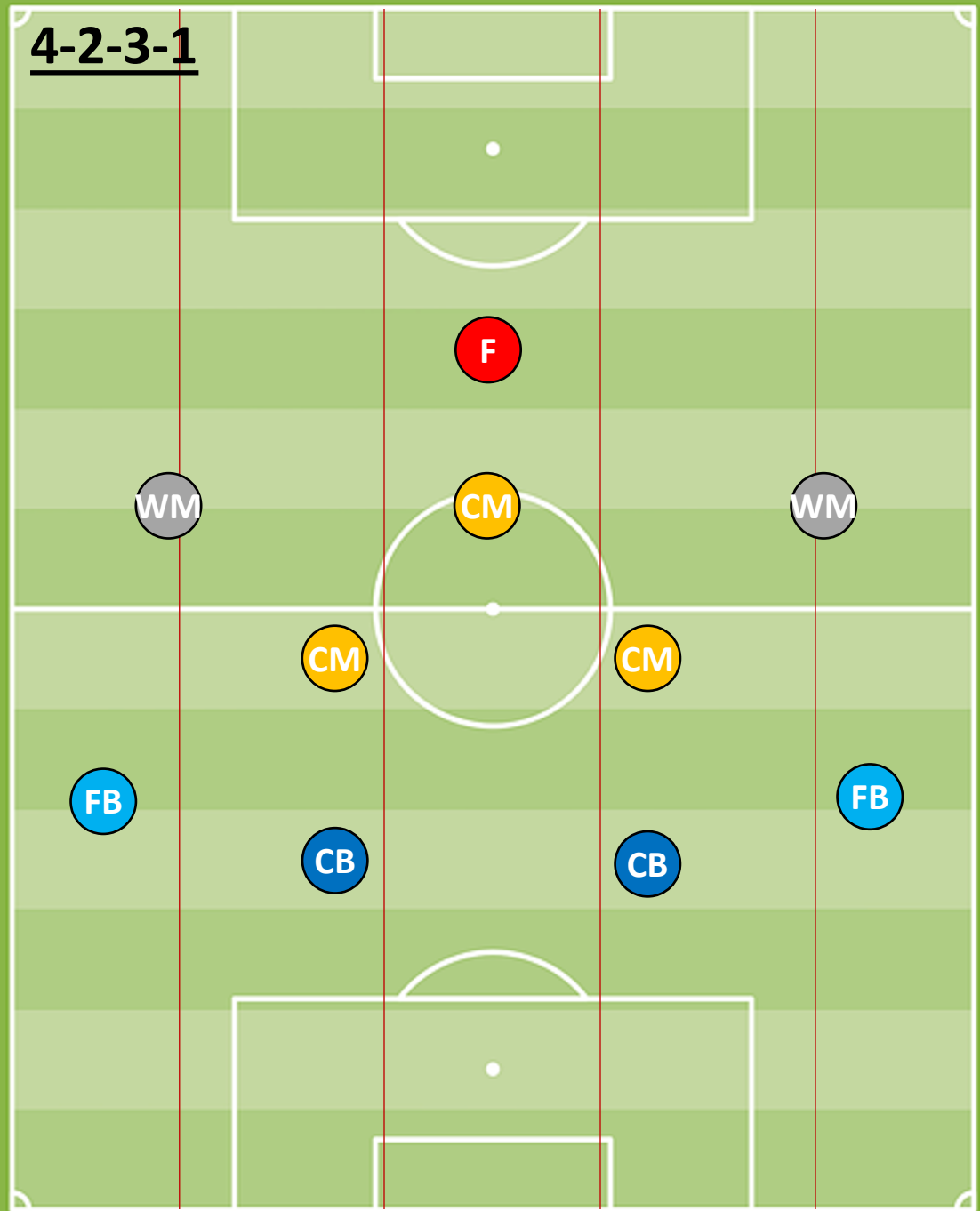

3-5-2

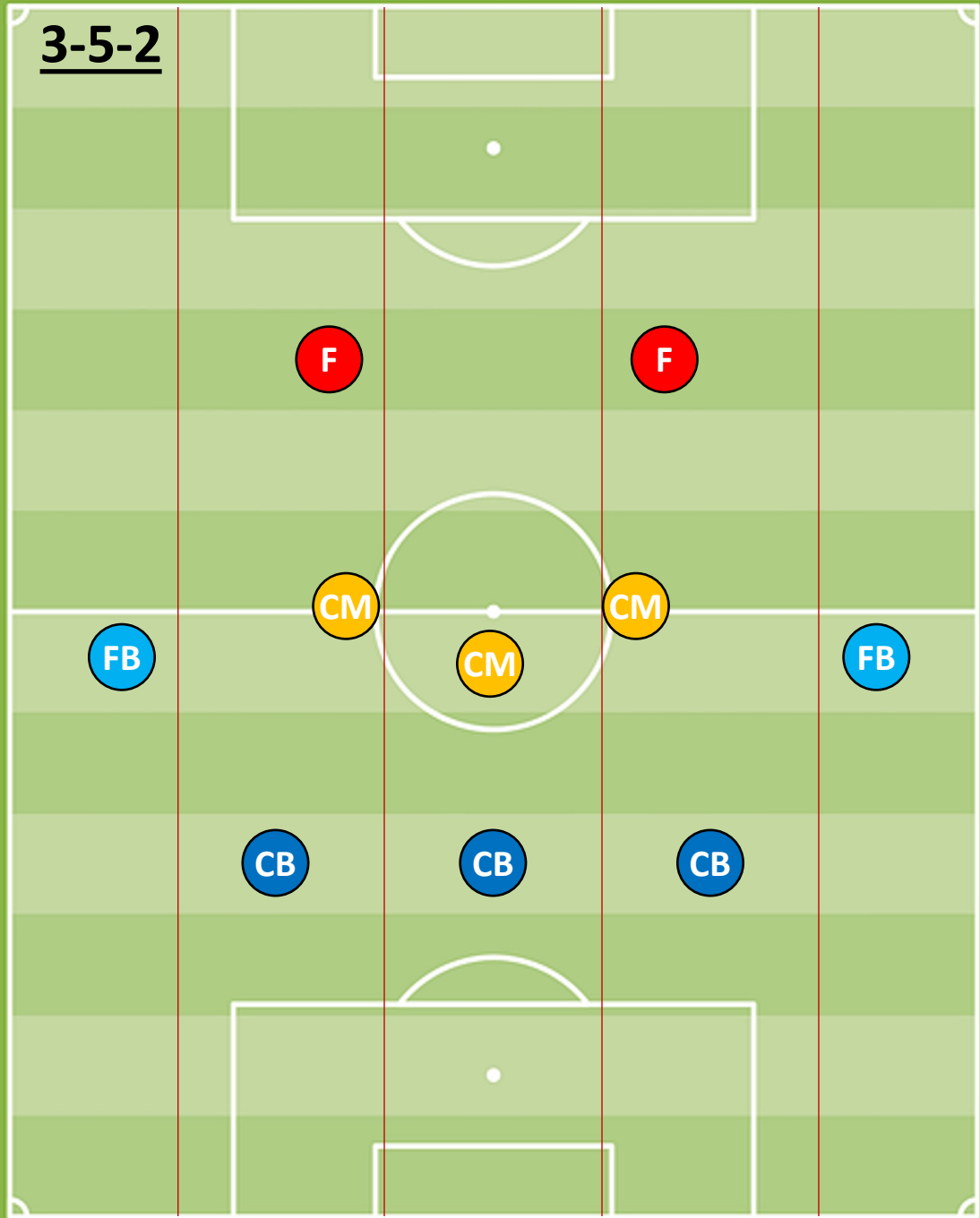

3-4-3

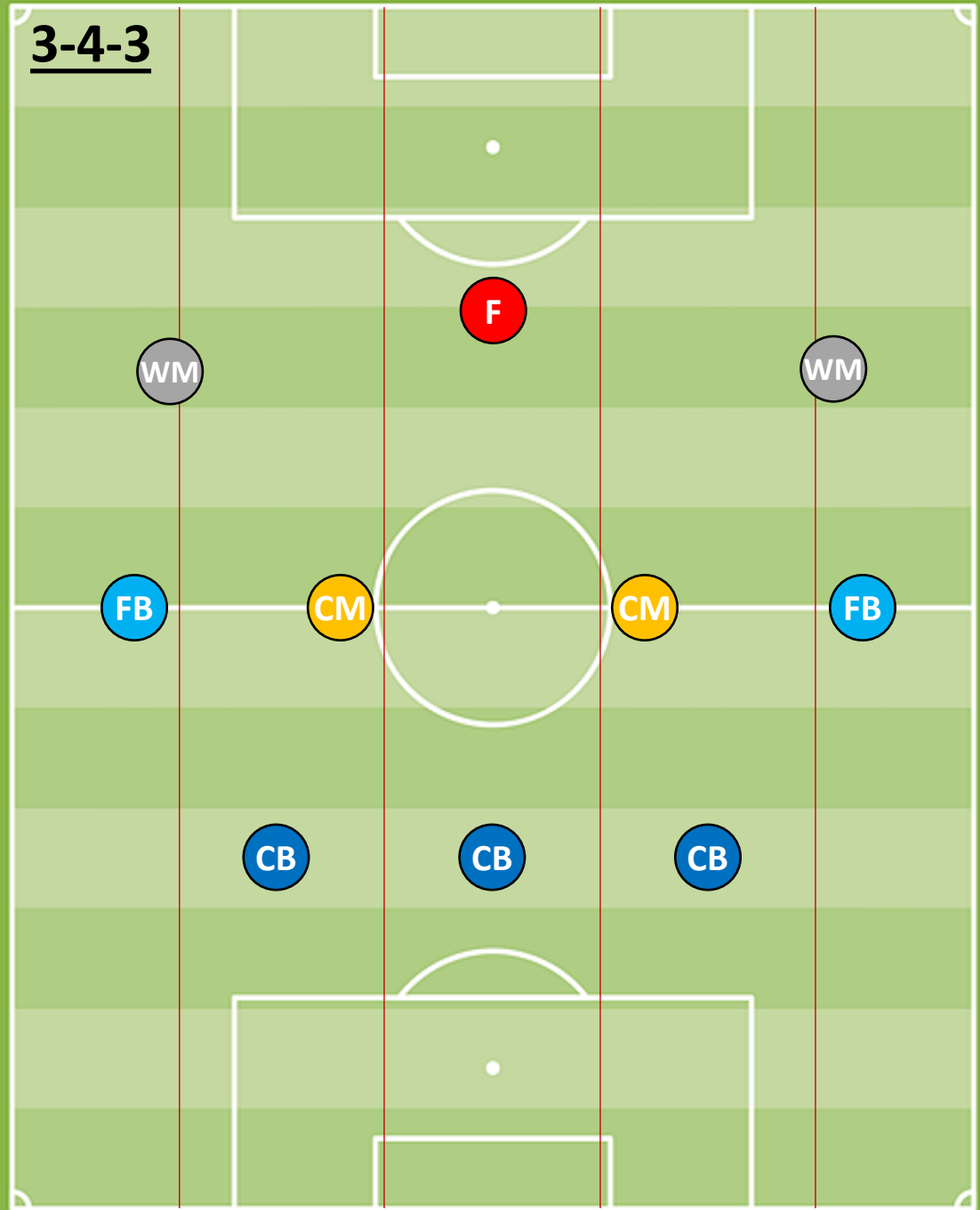

# 3-4-3 diamond

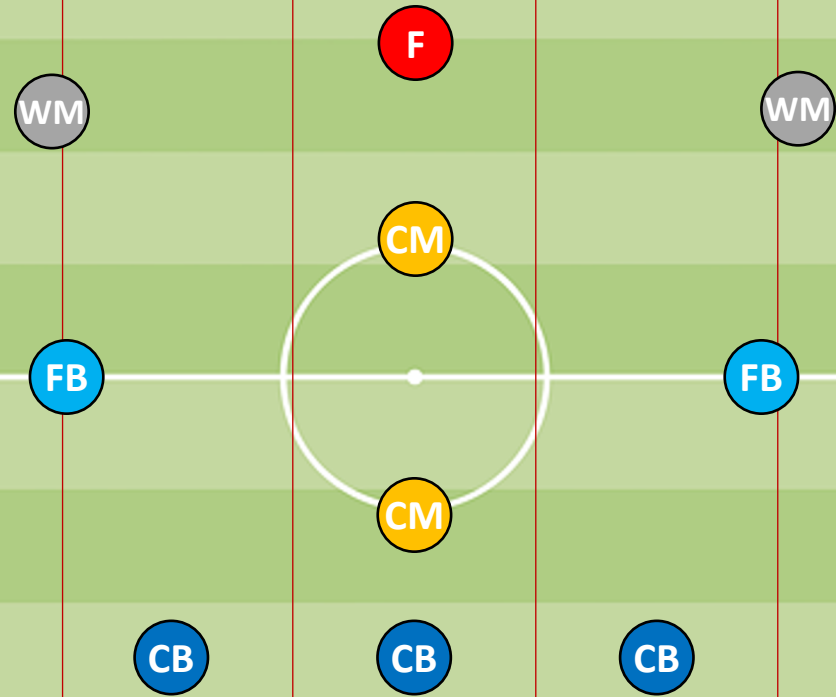

Supplement: S1 File — (PDF) [file pone.0265501.s005.pdf]
