# Supplementary material for: Center backs work hardest when playing in a back three: The influence of tactical formation on physical and technical match performance in professional soccer
Source: PLoS One. 2022 Mar 17;17(3):e0265501. doi: 10.1371/journal.pone.0265501 (PMC8929644; doi:10.1371/journal.pone.0265501)
Supplement: Attachment — Submitted filename: Response to Reviewers.docx [file pone.0265501.s006.docx]

**PONE-D-21-27534, entitled**

**"Center Backs Work Hardest When Playing in a Back Three: The Influence of Tactical Formation on Physical and Technical Match Performance in Professional Soccer"**

**Submitted to: PLOS ONE**

**Decision: Major Revisions**

**Point-by-point response**

Dear Editor,

Dear Reviewers,

The authors would like to thank the editor and the reviewers for their careful consideration and constructive criticism of this manuscript. We appreciate their positive comments, at the same time, agree with their suggestions for change, and have now revised our manuscript accordingly. Please find below a detailed point-by-point response.

The Authors

**JOURNAL REQUIREMENTS:**

1. When submitting your revision, we need you to address these additional requirements.

Please ensure that your manuscript meets PLOS ONE's style requirements, including those for file naming. The PLOS ONE style templates can be found at

<https://journals.plos.org/plosone/s/file?id=wjVg/PLOSOne_formatting_sample_main_body.pdf> and

<https://journals.plos.org/plosone/s/file?id=ba62/PLOSOne_formatting_sample_title_authors_affiliations.pdf>

*Thank you for this comment. We updated every aspect of the manuscript and file formatting that is required. (see page 1-19)*

2. We note that the grant information you provided in the ‘Funding Information’ and ‘Financial Disclosure’ sections do not match. When you resubmit, please ensure that you provide the correct grant numbers for the awards you received for your study in the ‘Funding Information’ section.

*We did not receive any third-party funds. Further, we updated the ‘Funding Information’ in the Cover letter.*

*Funding Statement: “The funder TSG 1899 Hoffenheim provided support in the form of salaries for authors [LeoF, TG]. The funder TSG ResearchLab gGmbH provided support in the form of salaries for authors [SA]. Both funders did not have any additional role in the study design, data collection and analysis, decision to publish, or preparation of the manuscript. The specific roles of these authors are articulated in the ‘author contributions’ section.”*

3. Thank you for stating the following in the Funding Section of your manuscript:

“The funder TSG 1899 Hoffenheim provided support in the form of salaries for authors [LF, TG]. The funder TSG ResearchLab gGmbH provided support in the form of salaries for authors [SA]. Both funders did not have any additional role in the study design, data collection and analysis, decision to publish, or preparation of the manuscript. The specific roles of these authors are articulated in the ‘author contributions’ section.”

We note that you have provided funding information that is not currently declared in your Funding Statement. However, funding information should not appear in the Acknowledgments section or other areas of your manuscript. We will only publish funding information present in the Funding Statement section of the online submission form.

Please remove any funding-related text from the manuscript and let us know how you would like to update your Funding Statement. Currently, your Funding Statement reads as follows:

“The funder TSG 1899 Hoffenheim provided support in the form of salaries for authors [LF, TG]. The funder TSG ResearchLab gGmbH provided support in the form of salaries for authors [SA]. Both funders did not have any additional role in the study design, data collection and analysis, decision to publish, or preparation of the manuscript. The specific roles of these authors are articulated in the ‘author contributions’ section.”

Please include your amended statements within your cover letter; we will change the online submission form on your behalf.

*We added the updated version of the ‘Funding Statement’ to the cover letter and excluded any funding information from the manuscript.*

4. Thank you for stating the following in the Competing Interests section:

“The authors have read the journal's policy and have the following competing interests to declare: The authors [LF, TG] were employed by the commercial affiliation TSG 1899 Hoffenheim. The authors [SA] were employed by the non-commercial limited liability company TSG ResearchLab gGmbH. This does not alter our adherence to PLOS ONE policies on sharing data and materials. There are no patents, products in development or marketed products associated with this research to declare.”

Please confirm that this does not alter your adherence to all PLOS ONE policies on sharing data and materials, by including the following statement: ""This does not alter our adherence to  PLOS ONE policies on sharing data and materials.” (as detailed online in our guide for authors <http://journals.plos.org/plosone/s/competing-interests>).  If there are restrictions on sharing of data and/or materials, please state these. Please note that we cannot proceed with consideration of your article until this information has been declared.

Please include your updated Competing Interests statement in your cover letter; we will change the online submission form on your behalf.

*We excluded any information about competing interests from the manuscript and added the updated version of the ‘Funding Statement’ to the cover letter.*

5. In your Data Availability statement, you have not specified where the minimal data set underlying the results described in your manuscript can be found. PLOS defines a study's minimal data set as the underlying data used to reach the conclusions drawn in the manuscript and any additional data required to replicate the reported study findings in their entirety. All PLOS journals require that the minimal data set be made fully available. For more information about our data policy, please see <http://journals.plos.org/plosone/s/data-availability>.

Upon re-submitting your revised manuscript, please upload your study’s minimal underlying data set as either Supporting Information files or to a stable, public repository and include the relevant URLs, DOIs, or accession numbers within your revised cover letter. For a list of acceptable repositories, please see <http://journals.plos.org/plosone/s/data-availability#loc-recommended-repositories>. Any potentially identifying patient information must be fully anonymized.

Important: If there are ethical or legal restrictions to sharing your data publicly, please explain these restrictions in detail. Please see our guidelines for more information on what we consider unacceptable restrictions to publicly sharing data: <http://journals.plos.org/plosone/s/data-availability#loc-unacceptable-data-access-restrictions>. Note that it is not acceptable for the authors to be the sole named individuals responsible for ensuring data access.

We will update your Data Availability statement to reflect the information you provide in your cover letter.

*We have indicated that the data from this study are available upon request because of legal restrictions by the data provider (DFL – Deutsche Fußball Liga). Requests to access the datasets should be directed to the DFL (*[*info@dfl.de*](mailto:info@dfl.de)*). The minimal data set we can provide can be found in the Supporting Information (S1 Table).*

6. We note that you have indicated that data from this study are available upon request. PLOS only allows data to be available upon request if there are legal or ethical restrictions on sharing data publicly. For more information on unacceptable data access restrictions, please see <http://journals.plos.org/plosone/s/data-availability#loc-unacceptable-data-access-restrictions>.

In your revised cover letter, please address the following prompts:

a) If there are ethical or legal restrictions on sharing a de-identified data set, please explain them in detail (e.g., data contain potentially sensitive information, data are owned by a third-party organization, etc.) and who has imposed them (e.g., an ethics committee). Please also provide contact information for a data access committee, ethics committee, or other institutional body to which data requests may be sent.

*We have indicated that the data from this study are available upon request because of legal restrictions by the data provider (DFL – Deutsche Fußball Liga).*

b) If there are no restrictions, please upload the minimal anonymized data set necessary to replicate your study findings as either Supporting Information files or to a stable, public repository and provide us with the relevant URLs, DOIs, or accession numbers. For a list of acceptable repositories, please see <http://journals.plos.org/plosone/s/data-availability#loc-recommended-repositories>.

We will update your Data Availability statement on your behalf to reflect the information you provide.

7. Please note that in order to use the direct billing option the corresponding author must be affiliated with the chosen institute. Please either amend your manuscript to change the affiliation or corresponding author, or email us at [plosone@plos.org](mailto:plosone@plos.org) with a request to remove this option.

*The corresponding author is affiliated with the chosen institute (see manuscript – title page).*

8. Please include your full ethics statement in the ‘Methods’ section of your manuscript file. In your statement, please include the full name of the IRB or ethics committee who approved or waived your study, as well as whether or not you obtained informed written or verbal consent. If consent was waived for your study, please include this information in your statement as well.

*We provided an ethics statement in the methods section. TO further help the understanding we changed this paragraph into the following: “Although data was collected as part of the players’ professional employment (22), ethical approval was obtained from the local ethics committee (Human and Business Sciences Institute, Saarland University, Germany, identification number: 22-02, 10 January 2022).” The cited article suggests that in the present setting (professional soccer players) an ethical approval is not required. (See page 5, lines 134-136)*

Reviewer #1: The authors studied how the tactical formation in soccer could affect different positions' technical and physical performance.

The manuscript is straightforward to read if the reader knows about soccer and statistical analysis. Maybe soccer's fans and specialized commentators are not always familiar with statistical analysis. Perhaps the curious scientific reader is not always familiar with soccer team formations like 4-4-2 or 5-3-2-1.

The recommendation is minor revisions:

Therefore, according to the previous paragraph, the suggestions are:

1) Make pictures to illustrate the different tactical formations, and identify the positions (for example, the "full back," "center back,"...). The graphical representation in soccer broadcasts and specialized websites is pretty good and could enhance the clarity.

*Thank you for the comment. We added images of the different tactical formations to the Supporting Information (See S5 File). In the added pictures the positions in the tactical formations become clear.*

2) The Statistical Analysis section could present more detailed information about the methods; in this case, keep in mind the soccer enthusiasts that may be not familiar with acronyms like "one-way ANOVA" or even a brief explanation about "mean" and "standard deviations."

*Thank you for the comment. To help the understanding of the statistical analysis we wrote out the word for ANOVA when it was first introduced in the manuscript. However, the ANOVA is a standard method in exercise science and should be familiar to the reader. Therefore, we act on the assumption that nearly all of the readers know what an ANOVA is. To explain the details of the statistical method would exceed the manuscript and would deviate from the topic of the study. (see page 6-7, lines 173-186)*

3) The data are previous to the Covid-19 pandemic. A meaningful change that occurred was the adoption of 5 substitutions per match, and there is the possibility that it becomes permanent, mainly due to the health concerns of the players. And this is a good point to discuss, mainly because this work could be compared (in the future) with this new scenario of 5 substitutions.

*Thank you for the comment. We added a paragraph to the limitation to address this problem and to indicate the problem for future studies. (see page 14, lines 377-380)*

Reviewer #2: Report of "Center Backs Work Hardest When Playing in a Back Three: The Influence of
3 Tactical Formation on Physical and Technical Match Performance in Professional
4 Soccer" by L. Forcher et al.

The manuscript above presents a study of whether tactical formation affects the physical and 42 technical match performance of professional soccer players in the first German Bundesliga. While the topic of the manuscript is interesting, there are certain aspects that need to be clarified further, specially the methodology and robustness of the findings, before this manuscript is suitable for publication. Please find my detailed points below.

1) My main concern is that the most important results were extracted using ANOVA software, which
provides limited information, specially to evaluate the robustness of the findings. It seems that the authors
used the ANOVA as a black-box without examining details in the calculations.

*Thank you for your comment and for sharing your concerns about the statistics. The ANOVA can be considered a robust method to detect arithmetic mean differences between groups. Further, the ANOVA is one of the most common methods to find significant group differences. Based on the studies mentioned underneath, the ANOVA is very robust against a violation of the pre-conditions (For example Blanca MJ, Alarcón R, Arnau J, Bono R, Bendayan R. Non-normal data: Is ANOVA still a valid option? Psicothema. 2017 Nov;29(4):552-557. doi: 10.7334/psicothema2016.383 . PMID: 29048317.). We suggest that the ANOVA as statistical method, is well studied and, therefore, should considered more likely a white-box than a black-box. In consideration of the huge number of groups and, therefore, the ANOVA represents the most robust choice in terms of statistical methods in our view. Moreover, to support the findings of the ANOVA we calculated effect sizes. The effect sizes can help the reader to interpret the differences that occurred in the ANOVA and help to interpret the robustness of the found results. In the limitations section, we discussed the problems and disatvantages with the ANOVA in the context of the present study and, therefore, ensure that the reader is capable of interpreting the results in the right way. We would be pleased, if you would share a deeper and more detailed version of your concerns. In order to further help the help the understanding of the reader we added a paragraph to the limitation section. (see page 15, lines 388-391)*

2) The manuscripts present several issues regarding the organization and final presentation. How do you cite? Did you put number or names in parentheses? Lines 70, 72 and 73 (#). Names, lines 155 and 156 (names). Capital letters: lines 191, 192 and 194, figure captions; figure, table,

*Thank you for making us aware of these issues. We corrected the citing and the formal aspects in the manuscript.*

3) In the introduction they do not say how the work is organized.

*In our field of study (sports science), it is not common to describe how the work is organized, as original investigations commonly follow a very similar pattern. The introduction sections should introduce the reader to the topic and deduce the main research question of the study. The scientific approach to address the research question is formally attributed to the methods section.*

4) The authors must consider additional metrics to present their results. The information provided by Deltatre can be used to explore additional properties, for instance, cross-correlations, etc.

*Cross-correlations concern the correlation of two signals, waves, or functions. After consultation with the research group, we are unsure how to implement this kind of statistical method into the study context. We would appreciate it if you could explain in detail how you would use cross-correlations in order to evaluate the present results. Furthermore, we would be pleased if you would name and explain other statistical methods that would help to provide other statistical results that can help to fix your concerns about the present statistical analysis.*

*In favor of the research question, we wanted to find the differences between different tactical formations. The correlations of the different tactical formations would analyze to what extent the performance of the players is equal/similar in the different formations.*

*Because we analyzed eight different tactical formations the results section is already very large. If we would use another statistical method to describe the effect of the tactical formation, we would expand this section. To the best of our knowledge, this would not provide any further information regarding the research question and would possibly downgrade the readability of the manuscript.*

5) The authors should also consider to incorporate methodologies like complex networks to reinforce their analysis and not just use conventional statistics.

*We suggested using a multi-level model to statistically evaluate our data. The mixed-model addresses inter- and intraindividual differences in the performance metrics. In favor of the research question, we wanted to exert the effect of the tactical formation and not of the individual player. Therefore, these statistical models would fit the dataset but not the research question. Therefore, we accepted and addressed the disadvantages of the ANOVA in order to properly answer the research question. We would appreciate it if you would provide more detailed suggestions in order to help us fix your concerns.*

6) As a general remark, it seems that they only make a statistical analysis but not checked or confirmed with anything else. The explanation of methods is not clear at all and it is not well understood the procedure. Also the quality of figures is very poor.

*We provided the figures in better quality to further help the readability of the manuscript. We tried to overhaul the statistics section in order to help the readability of the method section. . (see page 15, lines 388-391). We already addressed the pros and cons of the ANOVA. We would like to further explain to the Reviewer why we chose this statistical method. If we would consider the dependency of the groups (different tactical formations) and would suggest that the normal distribution is not given we would have to choose the Friedman-test to detect group-mean differences. Because we have eight execute different formations in ten different parameters for five different positions we have 1400 single tests. The ANOVA in SPSS controls for the alpha error with an alpha error correction (Bonferroni correction). We used Bonferroni post-hoc tests because they provide the most conservative and robust results in terms of alpha error correction. If we would execute every single group comparison independently the alpha error probability would increase significantly. Therefore, we stated the robust ANOVA with the disadvantages mentioned in the limitations as a better statistical method than dealing with a significantly increasing alpha error probability.*
